# Mouse Models of Gestational Diabetes Mellitus and Its Subtypes: Recent Insights and Pitfalls

**DOI:** 10.3390/ijms24065982

**Published:** 2023-03-22

**Authors:** Katharina Grupe, Stephan Scherneck

**Affiliations:** Institute of Pharmacology, Toxicology and Clinical Pharmacy, Technische Universität Braunschweig, Mendelssohnstraße 1, D-38106 Braunschweig, Germany; k.grupe@tu-braunschweig.de

**Keywords:** gestational diabetes mellitus, prediabetes, mouse model, GDM subtypes, impaired glucose tolerance

## Abstract

Gestational diabetes mellitus (GDM) is currently the most common complication of pregnancy and is defined as a glucose intolerance disorder with recognition during pregnancy. GDM is considered a uniform group of patients in conventional guidelines. In recent years, evidence of the disease’s heterogeneity has led to a growing understanding of the value of dividing patients into different subpopulations. Furthermore, in view of the increasing incidence of hyperglycemia outside pregnancy, it is likely that many cases diagnosed as GDM are in fact patients with undiagnosed pre-pregnancy impaired glucose tolerance (IGT). Experimental models contribute significantly to the understanding of the pathogenesis of GDM and numerous animal models have been described in the literature. The aim of this review is to provide an overview of the existing mouse models of GDM, in particular those that have been obtained by genetic manipulation. However, these commonly used models have certain limitations in the study of the pathogenesis of GDM and cannot fully describe the heterogeneous spectrum of this polygenic disease. The polygenic New Zealand obese (NZO) mouse is introduced as a recently emerged model of a subpopulation of GDM. Although this strain lacks conventional GDM, it exhibits prediabetes and an IGT both preconceptionally and during gestation. In addition, it should be emphasized that the choice of an appropriate control strain is of great importance in metabolic studies. The commonly used control strain C57BL/6N, which exhibits IGT during gestation, is discussed in this review as a potential model of GDM.

## 1. Introduction

GDM is a complex metabolic disorder and is defined as glucose intolerance with first recognition during pregnancy [1]. GDM is currently the most frequent complication of pregnancy with increasing prevalence. The prevalence of GDM varies considerably worldwide, ranging from 1% to more than 30% [2]. For instance, the prevalence in the United States was 8.2% in 2016 and had increased by 78% within 10 years [3]. It is challenging to compare prevalence in different countries and regions as there is a lack of consensus on uniform screening standards and diagnostic criteria [2]. Screening for GDM is recommended by the International Association of Diabetes and Pregnancy Study Group (IADPSG), the American Diabetes Association (ADA), and the Endocrine Society as a one-step 75 g oral glucose tolerance test (OGTT) at 24–28 weeks of pregnancy [4,5,6]. GDM has substantial risks and consequences for both mother and child. Consequences for the mother include, among others, increased risk of cesarean section, preeclampsia, and the likelihood of developing type 2 diabetes mellitus (T2DM) later in life. Fetal complications of GDM comprise neonatal hypoglycemia, stillbirth, macrosomia as well as an increased risk of obesity and T2DM during childhood and adolescence [7,8]. There is evidence that GDM and T2DM share a common pathophysiology. In particular, similar risk factors such as obesity, as well as a common genetic background, contribute to the development of both types of diabetes [9,10,11]. Diabetes is a global health concern and especially the number of T2DM patients is increasing dramatically. T2DM was formerly described as adult-onset diabetes, but the prevalence is increasing in children and adolescents. Reasons are multifactorial and include increasing childhood obesity, changes in diet and exercise, maternal obesity and diabetes, as well as other factors that are still not fully understood [12]. GDM represents pre-T2DM and is described as a chronic dysfunction characterized by increasing insulin resistance (IR) with decreasing islet cell compensation [13]. In addition to the increasing number of manifest T2DM in young adults, high rates of prediabetes can be observed as well. The term prediabetes describes patients with blood glucose levels not fulfilling the criteria for manifest diabetes, although being too high to be classified as physiological. Prediabetes is a risk factor for developing diabetes as well as cardiovascular disease [14]. The development of T2DM is an insidious process, often preceded by undiagnosed prediabetes [15].

Experimental models contribute significantly to the understanding of the molecular basis, pathogenesis, and therapeutic efficacy of drugs in a multifactorial disease like diabetes mellitus [16]. However, the use of animal models for the study of human diseases should not disregard the fact that the knowledge gained is not completely transferable and has to be verified in humans. Differences in generation times as well as basal and occasional blood glucose levels should be mentioned in relation to metabolic studies during gestation. The mouse is an ideal model organism since the genomes of mice and men are highly similar. This is of particular importance for diseases with a high degree of genetic components, such as diabetes mellitus [17].

This review aims to provide an overview of existing mouse models of GDM (already very well reviewed by Pasek et al. [18]). We searched PubMed for studies using the search terms “gestational diabetes AND animal model” and “gestational diabetes AND mouse model” and summarized the results. Model development is complicated by the heterogeneity of GDM patients and their division into subpopulations. For this reason, a new model is proposed that does not represent conventional GDM but does exhibit preconceptional prediabetes and IGT during gestation and therefore serves as a model of a subpopulation of GDM. Apart from selecting an appropriate model to study the disease, choosing the right control strain is essential. This review will furthermore outline and discuss the suitability of a commonly used control strain (C57BL/6N) as a potential GDM model.

## 2. Genetic Risk Factors of GDM

The pathogenesis of GDM is closely related to that of T2DM and a common genetic background can be observed in both disorders. This is due to a common family history and the association of GDM with an increased risk of developing T2DM later in life [19]. Based on this hypothesis, numerous genetic studies of GDM have attempted to reproduce these findings of a genetic association between T2DM and GDM [20]. Candidate gene studies have subsequently shown that certain genetic variants associated with diabetes in non-gravid populations are more common in women who develop GDM. In particular, variants of T2DM-related genes, such as *CDKAL1*, *CDKN2AB*, *IGF2BP2, IRS1, KCNJ11, KCNQ1, MTNR1B*, and *TCF7L2*, were observed more frequently in women with GDM. Genes associated with maturity-onset diabetes (MODY), such as *GCK* (MODY2 gene), are also associated with GDM. In particular, these identified genes are relevant for peripheral insulin sensitivity, regulation of insulin secretion, and β-cell function [21,22,23,24]. A list of genes commonly associated with GDM is shown in Table 1.

## 3. Subtypes of GDM

With the increasing levels of hyperglycemia outside of pregnancy, it is quite reasonable to assume that many cases diagnosed as GDM are actually undiagnosed pre-pregnancy hyperglycemia of varying severity [2]. This was already assumed by Harris in 1988. Thus, GDM and/or gestational IGT could represent an already pre-existing preconceptional disorder diagnosed during pregnancy [37]. Conventionally, GDM screening is performed in late pregnancy, between weeks 24 and 28 of gestation. Early-onset or diagnosed GDM (diagnosed < 20 weeks gestation), however, may be associated with even poorer pregnancy outcomes than late-onset or diagnosed GDM [23,38]. Preconceptional screening for glucose intolerance could provide early evidence of potential complications during pregnancy. In particular, women with known risk factors for GDM, such as advanced age, genetic predisposition, previous GDM, overweight or obesity, and cigarette consumption, could benefit from early screening and modification of dietary behavior and lifestyle prior to as well as during early pregnancy [39]. At present, a gap exists regarding research on normal and abnormal glucose metabolism in early pregnancy. Furthermore, there are no evidence-based criteria for diagnosing early-onset GDM [23,40,41].

According to conventional guidelines, GDM is considered a uniform group of patients. That differences between GDM patients exist was already studied and described by Cheney et al. in 1985. It was shown that lean GDM patients usually exhibit IGT due to a secretory defect, whereas obese patients show IR with hyperinsulinemia as well as decreased insulin receptor binding [42]. Heterogeneity of GDM has also been demonstrated in further studies, so classification into subpopulations seems reasonable [7,43,44,45,46]. Patients with a primary insulin secretion defect without impairment of insulin sensitivity and patients with a primary insulin sensitivity defect with hyperinsulinemia can be distinguished [43]. In addition, differences in obstetric and perinatal outcomes may exist between physiological GDM subtypes to some degree. Women with alterations in insulin sensitivity, for example, have a higher incidence of complications such as fetal macrosomia and cesarean section [23,47,48]. A better understanding of the phenotypic heterogeneity of the disease is needed in order to apply an individualized therapy. However, consistent methods for dividing GDM patients into different subtypes are lacking [49].

## 4. Conventional Mouse Models of GDM

Numerous animal models have been described in the literature for a better understanding of the pathomechanisms of GDM. Surgically and chemically induced animal models as well as dietary-induced and genetic manipulations are classified. These models, however, have certain limitations when investigating the pathogenesis of GDM [18]. As in other research areas, mice and rats are most commonly used as GDM models. In addition, pigs, sheep, dogs, as well as non-human primates, are used in metabolic research. Due of their size, these animals are particularly suitable for studies of glucose homeostasis after partial or total pancreatectomy [50,51,52,53]. However, the commonly used models of GDM lead to a non-physiological glycemic condition with severe and irreversible hyperglycemia. Therefore, the transient glucose tolerance disorder of GDM is not adequately reflected [54]. Genetic susceptibility is a critical factor in the development of GDM and numerous knockout models resembling the phenotype of GDM are described. A quite frequently used mouse strain in recent studies is the C57BL/KsJ^db/+^ mouse (*db/+*). Mice heterozygous for the leptin receptor develop moderate glucose intolerance during gestation and show increased weight gain compared to the wild-type strain, contributing to the development of IR and a GDM-like phenotype. Furthermore, regardless of fetal genotype, the offspring display macrosomia which is frequently observed in infants of GDM patients [55,56,57,58].

The prolactin receptor (PrlR) has an essential role in β-cell adaptation and its expression is induced during pregnancy [59,60]. The PrlR belongs to the type I cytokine receptor family and binding of prolactin or placental lactogen activates intracellular signaling cascades. Subsequently, transcription of proteins that regulate β-cell proliferation via the Janus kinase and signal transducer and activator of transcription (Jak2/Stat5) signaling pathway are induced [61,62]. Mice heterozygous for the PrlR (*PrlR^+/−^*) display a moderate glucose intolerance and decreased β-cell proliferation during gestation and serve as a model of GDM [18]. Furthermore, circulating maternal prolactin and placental lactogen induce the expression of the serotonin receptor (Htr2b) and tryptophan hydroxylases 1 and 2 (Tph1 and Tph2) during pregnancy, resulting in increased serotonin synthesis of the β-cell. Proliferation is regulated in an autocrine or paracrine manner after binding of serotonin to its receptor, but the exact mechanism is still poorly understood. Female Htr2b knockout mice (*Htr2b^−/−^*) develop a moderate glucose intolerance and exhibit reduced β-cell mass, as well as a decreased β-cell proliferation during gestation [18,63]. The transcription factor Foxm1, which regulates the transcription of cell cycle-related genes, additionally leads to increased β-cell proliferation during pregnancy. Pancreas-specific knockout of Foxm1 (*FoxM1^Δpanc^*) prevents the induction of maternal β-cell proliferation as well as the increase in β-cell mass, resulting in IGT that deteriorates as gestation progresses, contributing to the GDM phenotype [64,65].

Genetically manipulated mouse models of GDM exhibit characteristics of the human disease and single gene mutations are therefore useful for identifying pathways underlying GDM. However, these models cannot fully describe the heterogeneous spectrum of this polygenic disease [18]. Each of the existing models contributes to the study of the pathomechanisms of GDM. However, the heterogeneity of the disease, in particular the transient nature of human GDM, can thus still be inadequately reflected. Therefore, additional animal models of GDM are needed that display the full range and the transient nature of the disease. Furthermore, models that represent the different subpopulations of human GDM are needed which allow the identification of potential biomarkers for early diagnosis and the development of appropriate therapeutic treatment options.

## 5. The New Zealand Obese (NZO) Mouse—A Model of a Common Subpopulation of Human GDM

The NZO mouse is one of the most thoroughly studied polygenic mouse models of both human metabolic syndrome as well as T2DM [66]. The inbred strain, generated in 1948 by Marianne and Franz Bielschowsky at the University of Otago in New Zealand, was obtained by mating mice with an agouti coat color. As a result of the inbreeding, a large number of animals from the 10th generation onwards showed increased body weight, and from the 12th to the 17th generation they were selectively mated for this characteristic [67]. Obesity is partly due to hyperleptinemia in the presence of leptin resistance caused by an altered leptin receptor, resulting in hyperphagia [68]. Lower spontaneous activity in the NZO mouse leads to reduced energy consumption and thus contributes to the development of obesity [69]. Studies using this strain and the crossing of NZO mice with lean mouse strains, such as the Swiss Jim Lambert (SJL) or the C57BL/6 mouse strain, have identified diabetogenic and adipogenic gene variants [66]. It should be noted that these gene variants may originate from the lean mouse strain, therefore becoming active once the mice are rendered obese by crossbreeding with a polygenic strain such as the NZO [70,71,72]. The advantage of a polygenic mouse model compared to monogenic models is clearly shown as the phenotype is not expressed by an artificial knockout. Since diabetes mellitus is a polygenic disease in humans, NZO mice are a suitable model for the study of the pathophysiology [73]. Development of overt T2DM is limited to males in all known NZO sublines [66,74]. In contrast, female mice of this strain are protected from hyperglycemia and β-cell death partly due to the sex hormone estrogen [66]. Estrogen leads to protection against oxidative β-cell damage and apoptosis, which is mediated by the estrogen receptor-α [75]. Furthermore, estrogen is involved in the stimulation of hepatic fatty acid metabolism and suppression of hepatic glucose production [76]. However, the metabolic status of the female NZO mouse deteriorates after long-term administration of a high-fat diet or after ovariectomy. These animals develop a T2DM-like clinical phenotype resembling that of humans [77,78].

Since studies suggest that GDM and T2DM share a common pathophysiology, the characterization of female NZO mice as a potential model to study the pathophysiology of GDM seems reasonable. Female NZO mice show no overt hyperglycemia but exhibit IGT preconceptionally, whereas glucose tolerance does not deteriorate during gestation (day 14.5) and persists postpartum. Furthermore, NZO mice are characterized by preconceptional hyperinsulinemia and hyperglucagonemia, which slightly improves during gestation. NZO mice show an improved ability to stimulate insulin secretion during gestation, whereby this is insufficient to reverse the IGT. When determining the proliferative response of Langerhans islets to the increased insulin demand during gestation, NZO mice show a proliferation defect where proliferation remains almost unchanged compared to the preconceptional state. In contrast to the in vivo experiments, freshly isolated primary islets of the NZO strain show a weak secretory response in perifusion experiments both preconceptionally and on day 14.5 of gestation. Since the improvement of insulin secretion could not be observed ex vivo, it is reasonable to assume that modulators of insulin secretion such as pregnancy hormones and cytokines or the interaction of peripheral organs have an advantageous effect on insulin secretion and glucose homeostasis [79]. Studies have shown that serotonin (5-HT) is produced in islet cells during gestation and is involved in the maintenance of glucose homeostasis. The influence of placental lactogen on β-cells results in an upregulation of 5-HT synthesis, which contributes to increased insulin secretion and β-cell proliferation [80,81,82]. However, the effect of 5-HT on insulin secretion remains a matter of controversy [83,84,85]. Female NZO mice have increased plasma, pancreatic, and islet cell 5-HT concentrations and 5-HT gene expression but a decreased and glucose-independent 5-HT secretion. Moreover, an inhibitory effect of high 5-HT concentrations on glucose-stimulated insulin secretion, as well as on glucagon secretion, can be observed in this mouse strain. The increased 5-HT content in β-cells and the resulting paracrine inhibition of α-cells may account for the enhanced hyperglucagonemia during gestation [86]. In addition to the influences of 5-HT, 17β-estradiol (E2) was shown to be a modulator of glucose-stimulated insulin secretion. E2 levels increase gradually in mammals from the first to the third trimester and have a significant influence on carbohydrate, lipid, and intermediate metabolism. As a marker of insulin sensitivity, E2 could indirectly indicate a GDM phenotype [77,87]. NZO mice are characterized by elevated E2 levels during gestation and incubation with E2 results in increased glucose-stimulated insulin secretion, leading to the conclusion that E2 prevents deterioration of the metabolic state during gestation [88].

In addition to the effects of gestation on glucose homeostasis and pancreatic islet cell secretion, the effects of gestation on the liver and, in particular, on the lipid profile of the NZO mouse strain have been described. Metabolic adaptations during pregnancy include physiological IR and a slight increase in blood lipid concentration. These metabolic changes are required to meet the higher energy demands and thus ensure an adequate nutrient supply for the growth and development of the fetus [44,89,90]. This leads to an increase in plasma lipids, such as total cholesterol (TC), triglycerides (TG), and cholesterol in low-density lipoprotein (LDL-C) as well as very-low-density lipoprotein (VLDL-C) [91,92,93]. Furthermore, lipolytic hormones lead to an increase in free fatty acids (FFA) in late pregnancy, which contribute to physiological IR [94]. FFA are elevated in plasma in obesity, in prediabetes, and in T2DM as well as GDM and may therefore represent potential biomarkers [95,96,97]. Female NZO mice exhibit increased triglyceride, FFA, and sphingosine-1-phosphate (S1P) plasma levels during gestation. The decrease in the hepatic sphingomyelin reservoir associated with increased plasma S1P concentrations, which is a known marker of impaired lipid metabolism in diabetic rodent models, may have an inhibitory effect on insulin signaling and potentially mediate an increase in IR. Furthermore, female NZO mice show impaired hepatic weight adjustment and alterations in hepatic FFA metabolism during gestation [98]. To characterize the altered hepatic lipid metabolism of the NZO strain, hepatic fatty acid translocase (FAT/CD36) and its transcriptional regulator peroxisome proliferator-activated receptor alpha (PPARα) have been described. These proteins have a key role in the FFA metabolic pathway. PPARα is a transcriptional regulator of CD36, which is necessary for the uptake of FFA into hepatocytes [99,100]. Female NZO mice exhibit decreased PPARα expression within the liver and an absence of translocation of CD36 to the hepatocellular plasma membrane. This lack of CD36 translocation to the plasma membrane leads to reduced hepatic FFA uptake and increased plasma FFA concentrations [98]. Increased CD36 expression in the liver of male NZO mice is associated with higher production and accumulation of triglycerides and diacylglycerols. This leads to reduced hepatic insulin sensitivity and the subsequent onset of T2DM in male NZO mice [77].

NZO hepatocytes produce significantly higher amounts of glucose both preconceptionally and on day 14.5 of gestation. Moreover, NZO mice show a markedly diminished response to insulin, and glucose production fails to be suppressed by insulin. After preincubation with E2, hepatocytes show a marked response with decreased glucose production. A co-stimulation of insulin and E2 could not further reduce the glucose production in NZO hepatocytes. Moreover, pregnant NZO mice show reduced hepatic glycogen content, increased cyclic adenosine monophosphate (cAMP) levels, and reduced AKT activation as an indicator of insulin sensitivity. These differences were abolished after E2 stimulation. Thus, E2 seems to have protective effects on glucose metabolism both preconceptionally and during gestation. These results suggest that E2 protects the prediabetic phenotype of the NZO strain against metabolic deterioration and stabilizes GDM progression. Although this effect is not sufficient to fully restore the altered glucose metabolism in NZO mice, it might compensate for the increased hepatic IR. In addition to the effects of E2 on hepatic glucose metabolism, the influence of 5-HT was studied. 5-HT incubation increased hepatic glucose production and co-incubation with insulin reversed this 5-HT effect. Moreover, 5-HT decreased hepatic glucose uptake as well as the glycogen content. In conclusion, 5-HT has a deteriorating effect on hepatic glucose metabolism in NZO mice [86].

Although the preconceptional IGT is not consistent with the conventional GDM definition, female NZO mice display important characteristics of subpopulations of human GDM and/or prediabetes during gestation. This highlights the heterogeneity of the metabolic disease. Interestingly, the metabolic dysfunction occurring preconceptionally does not further deteriorate during gestation. Due to the preconceptionally elevated 5-HT plasma levels, this could be a predictive indicator for the GDM subtype with preconceptional IGT and may be suitable for early prediction of GDM risk. Consequently, the NZO model may contribute significantly to the characterization of patient cohorts with prediabetes prior to pregnancy that are diagnosed as GDM patients during pregnancy.

## 6. The C57BL/6N Mouse—A Model of Human GDM

One of the most frequently used mouse strains in research is the C57BL/6 inbred strain. Different substrains of the C57BL/6 mouse exist with the most commonly used lines being C57BL/6J and C57BL/6N [101,102]. Due of its susceptibility for developing diet-induced obesity (DIO), the C57BL/6J substrain is an established model in metabolic research [103]. This substrain carries a deletion of the nicotinamide nucleotide transhydrogenase (*Nnt*) gene which encodes a mitochondrial enzyme involved in NADPH synthesis [104]. The Nnt mutation has been associated with decreased glucose-stimulated insulin secretion and IGT [103,105]. Mice of this strain have been described as a model of GDM after feeding a high-fat diet or a high-fat-high-sugar diet [106,107].

The C57BL/6N strain exhibits both elevated blood glucose levels after 6 h of food deprivation and an IGT during gestation. Moreover, a deteriorating trend in glucose-stimulated insulin secretion was observed during gestation, which was accompanied by increased basal insulin concentrations [98]. It is already described that the C57BL/6 mouse strain may serve as a potential GDM model after feeding a high-fat diet. When fed a standard diet, only the C57BL/6J substrain showed an IGT in several studies, which is associated with the Nnt mutation [104,105,108]. Thus, the question arises whether the C57BL/6 mouse strain is an appropriate control strain in metabolic studies. Possible explanations of contrasting experimental results include different procedures for the determination of glucose tolerance as there is no consistency in the fasting duration (0, 3, 6, 24 h, and overnight fasting), the route of administration (intraperitoneal vs. oral) and the load of glucose given (3, 2, 1, or 0.5 mg/g). To study glucose tolerance, the following parameters are recommended: 2 mg glucose per g body weight administered orally after a 6 h fasting period. These parameters were evaluated by Andrikopoulos et al. and enabled robust results to study plasma glucose and insulin levels [79,98,109].

In addition, the C57BL/6N strain exhibits increased CD36 expression. Increased CD36 expression is observed in patients with non-alcoholic fatty liver disease and contributes significantly to dyslipidemia in mice [110,111]. Therefore, expression of CD36 might be a marker for metabolic deterioration during gestation [98]. Due to the IGT first occurring during gestation, the C57BL/6N mouse strain represents an adequate model of human GDM.

## 7. Choosing the Right Control for Metabolic Studies

The Naval Medical Research Institute (NMRI) outbred strain is an established control in diabetes and obesity research [112]. Using outbred strains has the advantage of genetic heterogeneity, like in humans. Due to multiple genetic variants, the outbred strain exhibits the entire allelic spectrum leading to a broad genetic variability [113,114]. Female NMRI mice exhibit increased body weight on a standard diet and are thus a suitable control for the obese NZO strain [79]. Compared to the C57BL/6 mouse, NMRI mice show reduced susceptibility to DIO [115]. Moreover, the NMRI strain is a metabolically healthy control, which shows an ordinary physiological adaptation during gestation and a robust β-cell physiology. NMRI mice show increased β-cell proliferation, islet size, and a slight increase in insulin content during gestation. In addition, female NMRI mice show a marked ability to stimulate insulin secretion and a physiological decrease in insulin sensitivity during gestation in the presence of the physiological stressor of a high litter size [79].

Pregnancy is a physiological model of an increase in pancreatic β-cell mass in which increased β-cell proliferation and hypertrophy occur as an adaptation to progressive IR [116,117]. An average two- to threefold increase in β-cell mass occurs in mice, primarily due to increased β-cell proliferation [118,119]. The two- to threefold increase in β-cell mass during gestation described in the literature could not be observed in the NMRI strain. Nevertheless, the strain exhibits both a significant increase in proliferation and increased plasma insulin concentrations, as well as insulin secretion during the oral glucose tolerance test [79]. The data indicate that increases in proliferation and insulin secretion are not necessarily accompanied by increases in β-cell mass. The increased insulin secretion in the absence of changes in β-cell mass could indicate enhanced β-cell functionality and secretion. Studies in rodents have shown that the two- to threefold increase in β-cell mass during gestation cannot account for the increased insulin secretion alone. Therefore, increased functionality of the β-cell with enhanced secretion can be assumed. The increase in glucose-stimulated insulin secretion could be due to a lowering of the threshold for glucose stimulation, which may be a consequence of increased expression of glucose transporter 2, glucokinase, and coupling between β-cells [119,120,121,122,123].

Furthermore, the NMRI outbred strain is an ideal model for the investigation of physiological insulin secretion. In particular, the triggering and amplifying pathway of insulin secretion is studied in more detail using this strain [124,125,126]. Consequently, the NMRI mouse is a recommended control strain for metabolic studies prior to and during gestation.

## 8. Conclusions

Numerous experimental models of GDM have been characterized in the literature to describe the pathomechanisms of the disease. In this regard, each model contributes to a better understanding of the effects of GDM on both the mother and the unborn child, although it remains a challenge to reflect all the underlying causes and consequences in humans. An overview of existing models of GDM and their strengths and weaknesses is given in Table 2.

Therefore, due to the heterogeneity of the disease, a single ideal GDM model may not be possible. The fact that different subtypes of GDM exist further challenges the generation of experimental models. Polygenic mouse models such as the NZO mouse could therefore be an adequate model, since different genetic variants contribute to the expression of a polygenic disease such as GDM. Although this mouse strain has limitations as well, it is suitable for characterizing GDM patients exhibiting IGT prior to pregnancy or displaying prediabetes, which is diagnosed as GDM during pregnancy. Since certain subpopulations are likely to be affected by a preconceptional disorder, it can be assumed that these patients could benefit from early screening. Early screening and intervention could contribute to the prevention of GDM and reduce the consequences for both mother and child. In addition to the NZO mouse, the C57BL/6N strain, commonly used as a control strain, emerged as a GDM model. Due to physiological differences between humans and animals, verification of findings obtained in animal models is necessary. Further studies are needed both to characterize the different subtypes of human GDM and to establish appropriate experimental models that represent these subtypes, allowing the study of the underlying pathomechanisms.

## Figures and Tables

**Table 1:** Genes associated with GDM.

Gene	Encoded Protein	Protein Function	Reference
*CDKAL1*	CDK5 regulatory subunit associated protein 1 like 1	regulation of β-cell function and glucose-stimulated insulin secretion; associated with impaired insulin secretory capacity	[23,25,26,27,28]
*CDKN2AB*	cyclin-dependent kinase inhibitor 2A/B	regulation of cell proliferation and apoptosis; control of glucose homeostasis, insulin secretion and β-cell function	[9,23,29,30]
*GCK*	glucokinase	phosphorylation of glucose in pancreatic β-cells and hepatocytes; involved in the regulation of insulin secretion; conversion to glycogen in the liver; MODY2 gene	[21,23,25,31]
*IGF2BP2*	insulin like growth factor 2 mRNA binding protein 2	modulates cellular metabolism by post transcriptional regulation; associated with impaired β-cell function	[22,23,32]
*IRS1*	insulin receptor substrate 1	important role in insulin signaling pathways	[22,31]
*KCNJ11*	potassium inwardly rectifying channel subfamily J member 11	encodes the inward-rectifier potassium ion channel (Kir6.2); regulation of insulin secretion	[21,31,33]
*KCNQ1*	potassium voltage-gated channel subfamily Q member 1	regulation of insulin secretion; associated with impaired β-cell function	[21,34]
*MTNR1B*	melatonin receptor 1B	regulation of insulin secretion; associated with impaired β-cell function	[21,22,35]
*TCF7L2*	transcription factor 7 like 2	transcription factor involved in WNT signaling pathway; associated with IR and impaired insulin secretion	[21,22,36]

**Table 2:** Overview of animal models of GDM.

Model/Strategy	Advantages	Disadvantages
Surgically induced by pancreatectomy	Suitable when other methods are not an option, especially for larger animals	Not accurately resembling the etiology of the human disease
	Studying the fetal development affected by changes in the uterine environment	Causes severe and irreversible hyperglycemia, not adequately reflecting the transient glucose intolerance of GDM
Chemically induced using Streptozotocin or Alloxan	Effective, affordable, time saving and widely used method	Not accurately resembling the etiology of the human disease Causes severe and irreversible hyperglycemia, not adequately reflecting the transient glucose intolerance of GDM
Nutritional manipulation: administration of a high-fat or high-fat high-sugar diet; glucose infusion	Suitable when other methods are not an option, especially for larger animals Similar to the human disease where obesity and lifestyle are major contributors	Disregards the genetic factors associated with the disease Leads to a condition similar to T2DM with a marked insulin resistance
Genetic manipulation	Investigation of β-cell adaptation mechanisms during gestation Studying the genetic mechanisms involved in human disease	Limited to specific animal models Large number of animals used for the generation of knockout models Inadequately reflects the complex interaction between polygenic and environmental factors
Polygenic models: e.g., New Zealand obese (NZO) mouse	Reflects the polygenic character of the disease Suitable for the characterization of a subpopulation of human GDM exhibiting IGT prior to pregnancy or prediabetes	Preconceptional IGT not consistent with the conventional GDM definition
Acquired model: C57BL/6N	Shows IGT during gestation in the absence of an intervention	Controversial model which is commonly used as a control strain

## Data Availability

Not applicable.
